# Improvements in quality of life and functional status in patients with psoriatic arthritis receiving anti–tumor necrosis factor therapies

**DOI:** 10.1002/acr.20104

**Published:** 2010-03

**Authors:** Amr A Saad, Darren M Ashcroft, Kath D Watson, Deborah P M Symmons, Peter R Noyce, Kimme L Hyrich

**Affiliations:** 1 School of Pharmacy and Pharmaceutical Sciences, University of ManchesterManchester, UK; 2 Arthritis Research Campaign Epidemiology Unit, University of ManchesterManchester, UK

## Abstract

**Objective:**

To evaluate the impact of anti–tumor necrosis factor (anti-TNF) therapies on quality of life (QOL) and functional status in psoriatic arthritis (PsA) patients and study potential predictors for QOL improvements.

**Methods:**

The study was based on a cohort of 596 PsA patients receiving anti-TNF therapies. Changes in functional status and QOL were assessed using the Health Assessment Questionnaire (HAQ) and Short Form 36 (SF-36) questionnaire on a 6-month basis. The Short Form 6D (SF-6D) was calculated as a utility score. Univariate and multivariate linear regression models were developed to examine potential predictors of QOL improvements at 6 months, using a range of demographic, baseline disease-specific, and therapeutic variables.

**Results:**

At 6 months, significant improvements in all SF-36 subscale scores were found, with the greatest percentage improvement from baseline related to physical role (113.8%; 95% confidence interval [95% CI] 102.6, 125.0). The percent improvement for the physical component scale was 53.2% (95% CI 44.5, 61.9) at 6 months, whereas that for the mental component scale was 16.9% (95% CI 14.7, 19.2). The mean ± SD SF-6D score was 0.58 ± 0.07 at baseline, and this improved to 0.63 ± 0.06 at 6 months. The median HAQ score at baseline was 1.88 (interquartile range [IQR] 1.38–2.25) for the entire cohort, and this improved to 1.25 (IQR 0.63–1.88) at 6 months. Improvements in Disease Activity Score in 28 joints at 6 months were found to be significantly associated with QOL improvements at the same time point.

**Conclusion:**

Anti-TNF therapy is associated with improvement in both physical and mental status in PsA patients. These improvements were most substantial in patients who also had improvements in their disease activity.

## INTRODUCTION

Psoriatic arthritis (PsA) is a chronic inflammatory condition occurring in 0.2–1% of the general population (1,2) and 6–39% of patients with psoriasis (3–5). Both the joint and skin components of the disease have a profound impact on the quality of life (QOL) of patients with PsA (2,6), resulting in considerable physical and psychosocial morbidity (7,8). Daily symptoms of fatigue, pain, stiffness, and physical disability are common features in PsA for many patients (9). Persistent active disease without effective treatment may lead to permanent loss of physical function, reduced productivity, and increased rates of work disability (10). Skin involvement is also associated with a significant emotional burden that negatively impacts patients' QOL (11).

The impact of anti–tumor necrosis factor (anti-TNF) therapies (etanercept, infliximab, and adalimumab) on QOL has been studied in PsA in a number of randomized controlled trials (RCTs) (12–17) and recent longitudinal observational studies (18,19). However, the RCTs have only compared individual anti-TNF therapies against placebo, whereas the observational studies have either included no comparator (19) or compared treatment response against methotrexate (MTX) (18). To date, to our knowledge there is no study assessing which factors are important for predicting QOL improvements in PsA patients in routine clinical practice. This longitudinal observational study, therefore, aimed to monitor the impact of anti-TNF therapies on QOL and functional status of PsA patients, and to identify which factors (demographic or clinical) were associated with QOL improvements.

## PATIENTS AND METHODS

### Setting

The British Society for Rheumatology Biologics Register (BSRBR) was established in October 2001. This multicenter, longitudinal, observational study aims to monitor the safety and efficacy of biologic therapies in patients with inflammatory arthropathies in the UK (20). Although it is primarily a study of patients with rheumatoid arthritis (RA), the study also collected data on patients starting anti-TNF therapies for PsA between 2002 and 2006.

### Subjects and treatments

Subjects included in this study were those included in the BSRBR with a physician diagnosis of PsA starting 1 of 3 available anti-TNF agents (etanercept, infliximab, and adalimumab). The British Society for Rheumatology (BSR) guidelines for the use of anti-TNF therapies in PsA, published in February 2005, recommend that anti-TNF drugs should be reserved for patients with active PsA (defined as ≥3 tender joints and ≥3 swollen joints), despite adequate therapeutic trials of at least 2 standard disease-modifying antirheumatic drugs (DMARDs) individually or in combination (21). During the study, etanercept was administered as a subcutaneous injection of 25 mg twice weekly or 50 mg once weekly (22); adalimumab was administered as a subcutaneous injection of 40 mg every 2 weeks (23). The licensed dose of infliximab is 5 mg/kg administered at weeks 0, 2, 6, and 8, and then every 8 weeks thereafter. It is also recommended that infliximab be administered in combination with MTX (24).

### Data collection

At the time of initiation of the biologic drug, details of the patient's age, sex, diagnosis, disease duration, and current disease activity (using the 28-joint count Disease Activity Score [DAS28]) (25) were recorded by the consultant or rheumatology nurse. Details of past and present antirheumatic therapies and current comorbidities were also recorded. Each patient also provided details about current work status and ethnicity, and completed the Health Assessment Questionnaire (HAQ) adapted for British use (26) and the Short Form 36 (SF-36) health survey (27).

Rheumatologists and patients were each sent a 6-month postal followup questionnaire. Rheumatologists recorded current disease activity (DAS28), while patients completed the HAQ and SF-36. When questionnaires were not returned, reminders were sent to the rheumatologists after 5 weeks and to the patients after 2 weeks. Following a second period of 2 weeks of patient nonresponse, they were then sent another patient followup questionnaire.

The study was approved by the North West National Health Service Multicentre Research Ethics Committee and all of the subjects gave their written consent for participation.

### Statistical analysis

The primary outcome measure was the change in SF-36 scores (physical component scale [PCS] and mental component scale [MSC]) at 6 months. Secondary outcomes included the change in SF-36 scores between baseline to 12 months and 18 months, as well as the change in the 8 individual component scales at each time point.

The Short Form 6D (SF-6D) is a utility score derived from SF-36 ratings that was computed according to a published algorithm (28). It is formed from 11 items included in the SF-36, which are constructed to form 6 dimensions: physical functioning, role limitations, social functioning, pain, mental health, and vitality (28). Each of these 6 dimensions has between 4 and 6 possible levels. An SF-6D health state is defined by selecting one level from each dimension, through which 18,000 different health states can be defined. Level 1 in each dimension represents no loss of health or functioning in that dimension; thus, a state of “111111” indicates perfect health. In contrast, the worst possible state is “645 655.” The patient's current health state is then valued against the best and the worst possible health states on a 0–1 scale (based on a UK population), where 0 is the equivalent to being dead and 1 is the equivalent to perfect health.

Physical function was assessed using the change in the HAQ score at 6, 12, and 18 months. Paired *t*-tests (for the SF-36 and SF-6D) and Wilcoxon's signed rank test (for the HAQ) were performed to examine differences in response between baseline and followup results (at 6, 12, and 18 months) for the cohort as a whole and within each anti-TNF treatment cohort. Analysis of covariance (ANCOVA) was used to identify differences between the 3 anti-TNF therapy cohorts adjusting for age, sex, and baseline values of the HAQ and SF-36 scores. SF-36 PCS, SF-36 MCS, SF-6D, and HAQ scores were computed using 2 approaches: 1) using all available data at each followup, and 2) imputation of any missing data at each followup, assuming that data were missing at random, and predicted using previous scores, patient demographic details, and disease-specific and therapeutic variables (29,30).

Univariate and multivariate linear regression models were used to identify factors associated with changes in SF-36 scores at 6 months from baseline (31). Separate models were developed for change in the SF-36 PCS and SF-36 MCS. The following covariates were examined in the models: baseline demographic variables (age [years], sex, whether the patient had additional baseline comorbidities [yes/no], work status), baseline disease-specific variables (high inflammatory markers [C-reactive protein level >20 mg/liter and/or erythrocyte sedimentation rate >28 mm/hour], 28 tender joint count, and 28 swollen joint count), HAQ score, disease duration (years), treatment response (improvements in the DAS28 at 6 months), and therapeutic variables (anti-TNF therapy used and concurrent use of DMARDs or steroids [yes/no]). In the multivariate analyses, we used the 6-month improvement in the composite DAS28 score as a potential predictor rather than its individual components at baseline. The results are presented as β coefficients with corresponding 95% confidence intervals (95% CIs). All calculations were performed using Stata, version 9.0 (StataCorp, College Station, TX).

## RESULTS

### Demographic characteristics

A total of 596 patients with PsA were registered with the BSRBR between 2002 and 2006 (333 etanercept, 171 infliximab, and 92 adalimumab). Baseline characteristics of the PsA patients are shown in Table 1. The mean ± SD age was 45.7 ± 11.1 years, 53% were women, and the mean ± SD disease duration was 12.4 ± 8.7 years. The median HAQ score was 1.9 (interquartile range [IQR] 1.4–2.3), and the mean ± SD values for the PCS and MCS of the SF-36 were 19.1 ± 9.9 and 41.7 ± 11.6, respectively. There was no significant statistical difference between the 3 anti-TNF cohorts' demographic characteristics, functional status scores, or QOL ratings at baseline (Table 1).

**Table 1:** Demographic, functional status, and quality of life characteristics of patients with psoriatic arthritis at baseline*

	All (n = 596)	Etanercept (n = 333)	Infliximab (n = 171)	Adalimumab (n = 92)	*P*†
Demographic characteristics					
Age, years	45.7 ± 11.1	45.8 ± 11.1	44.8 ± 11.0	47.0 ± 11.6	0.325
Women, no. (%)	313 (52.5)	170 (51.1)	94 (55.0)	49 (53.3)	0.581
Disease duration, years	12.4 ± 8.7	12.8 ± 9.0	12.2 ± 8.0	11.4 ± 8.4	0.384
Working status, no. (%)‡					0.927
Working	245 (41.1)	135 (40.5)	67 (39.2)	43 (46.7)	
Unemployed but seeking work	3 (0.5)	2 (0.6)	1 (0.6)	0 (0.0)	
Not working due to ill health/disability	146 (24.5)	87 (26.1)	44 (25.7)	15 (16.3)	
Retired	70 (11.7)	38 (11.4)	16 (9.4)	16 (17.4)	
Functional status and quality of life characteristics					
Inflammation, no. (%)§	266 (44.6)	141 (42.3)	85 (49.7)	40 (43.5)	0.143
DAS28	6.4 ± 5.6	6.1 ± 1.2	7.3 ± 10.1	6.0 ± 1.0	0.464
Patient global assessment (100-mm VAS)	71.4 ± 21.1	71.5 ± 20.8	71.1 ± 23.0	71.5 ± 19.0	0.917
HAQ score, median (IQR)	1.9 (1.4–2.3)	1.8 (1.4–2.3)	2.0 (1.4–2.4)	1.8 (1.1–2.3)	0.581
SF-36 PCS	19.14 ± 9.94	18.99 ± 9.93	18.11 ± 9.59	21.19 ± 10.32	0.099
SF-36 MCS	41.73 ± 11.58	41.76 ± 11.55	40.33 ± 10.95	44.43 ± 12.53	0.052
SF-6D	0.58 ± 0.07	0.58 ± 0.06	0.57 ± 0.07	0.59 ± 0.06	0.052

* Values are the mean ± SD unless otherwise indicated. DAS28 = Disease Activity Score in 28 joints; VAS = visual analog scale; HAQ = Health Assessment Questionnaire; IQR = interquartile range; SF-36 = Short Form 36 questionnaire; PCS = physical component scale; MCS = mental component scale; SF-6D = Short Form 6D.

† *P* values are for statistical differences between the 3 anti–tumor necrosis factor cohorts at baseline.

‡ Working status was only available for 77.8% of the patients.

§ C-reactive protein level >20 mg/liter or erythrocyte sedimentation rate >28 mm/hour.

### QOL

Mean ± SD values for the PCS and MCS of the SF-36 are shown in Table 2. The largest changes were achieved within the first 6 months of treatment and were largely sustained throughout the followup period to 18 months. For the entire cohort, the mean ± SD PCS score improved from 19.1 ± 9.9 at baseline to 29.3 ± 13.7 at 6 months, whereas the mean ± SD MCS score improved from 41.7 ± 11.6 to 48.8 ± 11.7 at the same time points. The mean ± SD SF-6D score was 0.58 ± 0.07 at baseline and 0.63 ± 0.06 at 6 months. Imputing missing variables did not affect the magnitude of the QOL improvements (Table 2).

**Table 2:** Scores of quality of life and functional status in patients with psoriatic arthritis*

	Available data at each followup	Imputation of missing values†
SF-36 PCS		
Baseline (n = 510)	19.14 ± 9.94	19.14 ± 9.94
6 months (n = 400)	29.32 ± 13.69	28.27 ± 12.19
12 months (n = 363)	29.12 ± 13.24	28.52 ± 12.32
18 months (n = 317)	29.34 ± 13.91	27.59 ± 12.68
SF-36 MCS		
Baseline (n = 510)	41.73 ± 11.58	41.73 ± 11.58
6 months (n = 400)	48.79 ± 11.67	47.86 ± 11.12
12 months (n = 363)	47.74 ± 11.64	47.20 ± 11.23
18 months (n = 317)	48.58 ± 11.95	47.52 ± 11.57
SF-6D		
Baseline (n = 510)	0.58 ± 0.07	0.58 ± 0.07
6 months (n = 400)	0.63 ± 0.06	0.62 ± 0.05
12 months (n = 363)	0.63 ± 0.06	0.62 ± 0.06
18 months (n = 317)	0.63 ± 0.07	0.63 ± 0.06
HAQ score, median (IQR)		
Baseline (n = 562)	1.88 (1.38–2.25)	1.88 (1.38–2.25)
6 months (n = 424)	1.25 (0.63–1.88)	1.27 (0.65–1.89)
12 months (n = 382)	1.38 (0.63–2.00)	1.39 (0.64–2.01)
18 months (n = 344)	1.38 (0.63–2.00)	1.39 (0.64–2.01)

* Values are the mean ± SD unless otherwise indicated. SF-36 = Short Form 36; PCS = physical component scale; MCS = mental component scale; SF-6D = Short Form 6D; HAQ = Health Assessment Questionnaire; IQR = interquartile range.

† N = 510 for quality of life scores and n = 562 for physical function score.

The changes from baseline to 6 months were similar for all 3 anti-TNF agents. For the SF-36 PCS, the mean ± SD improvements were from 18.9 ± 9.9 to 29.4 ± 13.7, from 18.1 ± 9.6 to 27.7 ± 14.1, and from 21.2 ± 10.3 to 31.6 ± 12.8 for etanercept, infliximab, and adalimumab, respectively. Likewise, mean ± SD improvements from baseline to 6 months for the SF-36 MCS were 41.8 ± 11.5 to 48.7 ± 12.2 for etanercept, 40.3 ± 10.9 to 48.6 ± 10.9 for infliximab, and 44.4 ± 12.5 to 49.2 ± 11.4 for adalimumab. The mean ± SD SF-6D scores were 0.58 ± 0.06, 0.57 ± 0.07, and 0.59 ± 0.06 at baseline, respectively, and they were 0.63 ± 0.06, 0.62 ± 0.07, and 0.64 ± 0.06 at 6 months for the abovementioned 3 anti-TNF cohorts, respectively. There were no significant statistical differences in the percent improvement achieved among the 3 cohorts throughout the followup after adjusting for age, sex, and baseline values. These improvements were maintained throughout the followup to 18 months for the 3 cohorts.

**Figure 1:**
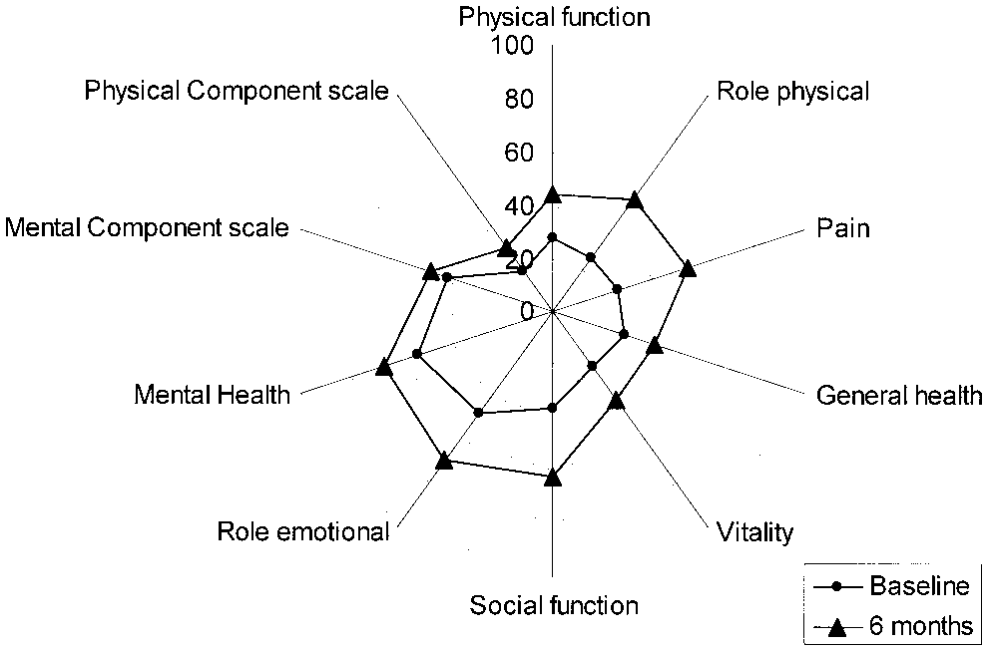
Spider plot of the mean scores at baseline and 6 months in the Short Form 36 components for patients with psoriatic arthritis receiving anti–tumor necrosis factor therapies.

At 6 months, the greatest improvements within the SF-36 instrument from baseline for the entire cohort were in the physical role component (where it improved from mean ± SD 24.3 ± 26.3 to 52.0 ± 31.1), followed by the pain component (mean ± SD 26.1 ± 19.8 at baseline and 53.6 ± 26.8 at 6 months), whereas the smallest improvements were found in the mental health component (improved from mean ± SD 53.7 ± 20.7 to 66.7 ± 21.1), as shown in Figure 1. The mean ± SD values of the 8 domains of the SF-36 for the PsA cohort over the 18-month followup period are detailed in Table 3. ANCOVA tests showed no significant statistical differences in the percent improvement achieved among the 3 cohorts throughout the followup period after adjusting for age, sex, and baseline values (data not shown).

**Table 3:** Values of the 8 domains of the Short Form 36 in patients with psoriatic arthritis at different followup times*

	Baseline	6 months	12 months	18 months
Physical function	27.43 ± 23.61 (537)	43.91 ± 29.16 (407)	42.56 ± 29.33 (377)	44.36 ± 29.59 (343)
Role (physical)	24.33 ± 26.34 (541)	52.01 ± 31.05 (430)	51.30 ± 31.52 (379)	53.65 ± 31.23 (344)
Bodily pain	26.09 ± 19.83 (550)	53.62 ± 26.79 (436)	53.23 ± 26.99 (390)	52.58 ± 27.14 (351)
General health	28.44 ± 18.26 (543)	39.96 ± 22.43 (420)	39.89 ± 21.89 (378)	40.99 ± 22.21 (344)
Vitality	25.75 ± 20.06 (541)	40.79 ± 22.82 (430)	40.47 ± 23.09 (383)	40.72 ± 23.42 (352)
Social function	36.43 ± 25.22 (546)	62.16 ± 28.75 (427)	60.58 ± 29.05 (390)	62.15 ± 28.89 (350)
Role (emotional)	47.47 ± 35.97 (546)	69.07 ± 30.51 (429)	67.12 ± 31.02 (385)	69.35 ± 30.97 (346)
Mental health	53.68 ± 20.72 (540)	66.66 ± 21.11 (428)	65.11 ± 22.13 (383)	67.27 ± 22.18 (353)

* Values are the mean ± SD (no.). *P* < 0.05 was calculated for each followup versus baseline within the same cohort.

### Functional status

The median HAQ score at baseline was 1.88 (IQR 1.38–2.25) for the entire cohort, and this improved to 1.25 (IQR 0.63–1.88) at 6 months (*P* < 0.001) (Table 2). Imputing missing variables did not affect the magnitude of the improvements observed for physical function. The median HAQ score improved from 1.75 (IQR 1.38–2.25) at baseline to 1.38 (IQR 0.50–1.88) at 6 months in the etanercept cohort, from 2.00 (IQR 1.38–2.38) at baseline to 1.25 (0.63–2.00) at 6 months in the infliximab cohort, and from 1.75 (IQR 1.13–2.25) at baseline to 1.19 (0.63–1.88) at 6 months in the adalimumab cohort. These responses were generally maintained for 18 months. There were no significant statistical differences in the improvements in HAQ score achieved among the 3 anti-TNF cohorts throughout the followup after adjusting for age, sex, and baseline values.

### Factors associated with QOL improvements at 6 months

Table 4 shows the results from the univariate and multivariate analyses examining predictors of changes from baseline in the SF-36 PCS and SF-36 MCS. For the change in the SF-36 PCS, the univariate analyses suggested that for each year increase in the patient's age, there was a lower improvement in the SF-36 PCS values (β = −0.14; 95% CI −0.25, −0.04). Patients who were not working due to ill health or disability (β = −5.34; 95% CI −8.05, −2.63) and those who were retired (β = −5.11; 95% CI −9.34, −0.88) had significantly lower SF-36 PCS values at 6 months compared with patients who were working. However, the presence of inflammation was associated with a greater improvement in the SF-36 PCS (β = 3.15; 95% CI 0.75, 5.55), as did a higher baseline swollen joint count (β = 0.30 per additional swollen joint; 95% CI 0.09, 0.51). In addition to baseline factors, improvements in DAS28 scores at 6 months were also strongly associated with improvements in SF36 PCS scores from baseline (β = 3.09 per unit improvement in DAS28; 95% CI 2.37, 3.81).

**Table 4:** Univariate and multivariate regression analysis of potential predictors and factors associated with changes in the PCS and MCS of the SF-36 at 6 months*

	Change in SF-36 PCS		Change in SF-36 MCS	
	Univariate	Multivariate	Univariate	Multivariate
Demographic variables				
Age at start of therapy, years	−0.14 (−0.25, −0.04)†	−0.02 (−0.17, 0.14)	0.08 (−0.03, 0.19)	0.09 (−0.10, 0.28)
Women	−1.73 (−4.06, 0.60)	0.67 (−2.10, 3.44)	0.01 (−2.46, 2.47)	0.95 (−2.34, 4.24)
Baseline comorbidity (yes/no)‡	0.40 (−2.11, 2.92)	0.19 (−2.68, 3.05)	−0.96 (−3.63, 1.72)	−1.99 (−5.39, 1.41)
Working status (working is the reference group)				
Unemployed but seeking work	10.37 (−4.82, 25.57)	7.94 (−6.93, 22.83)	13.26 (−2.97, 29.49)	7.15 (−10.52, 24.83)
Not working due to ill health/ disability	−5.34 (−8.05, −2.63)†	−4.64 (−7.71, −1.58)†	1.33 (−1.56, 4.22)	2.44 (−1.20, 6.08)
Retired	−5.11 (−9.34, −0.88)†	−4.49 (−9.52, 0.55)	−1.14 (−5.66, 3.38)	0.39 (−5.59, 6.38)
Disease variables				
Baseline HAQ score	−0.13 (−1.38, 1.12)	−0.22 (−1.51, 1.08)	0.99 (−0.34, 2.31)	−0.34 (−1.88, 1.20)
Disease duration, years	0.07 (−0.08, 0.21)	−0.09 (−0.26, 0.08)	0.18 (0.03, 0.34)†	0.10 (−0.10, 0.30)
Baseline inflammation§	3.15 (0.75, 5.55)†	–	2.07 (−0.49, 4.62)	–
Baseline tender joint count	0.10 (−0.06, 0.26)	–	0.01 (−0.16, 0.17)	–
Baseline swollen joint count	0.30 (0.09, 0.51)†	–	0.21 (−0.02, 0.43)	–
Treatment response				
Improvement in DAS28 at 6 months	3.09 (2.37, 3.81)†	2.92 (2.10, 3.75)†	3.09 (2.37, 3.81)†	1.31 (0.33, 2.29)†
Therapeutic variable: concurrent use				
DMARDs	1.61 (−0.76, 3.98)	2.07 (−0.79, 4.93)	1.03 (−1.49, 3.56)	−0.56 (−3.96, 2.84)
Steroids	1.43 (−1.57, 4.42)	1.60 (−1.46, 4.66)	3.77 (0.52, 7.02)†	2.38 (−1.25, 6.02)
Biologic therapy (etanercept is the reference category)				
Infliximab	−2.21 (−4.94, 0.54)	0.93 (−2.50, 4.35)	−1.02 (−3.94, 1.90)	2.54 (−1.53, 6.61)
Adalimumab	0.25 (−3.04, 3.54)	0.08 (−4.19, 4.36)	−1.97 (−5.49, 1.54)	3.88 (−1.19, 8.95)

* Values are the β coefficient (95% confidence interval). PCS = physical component scale; MCS = mental component scale; SF-36 = Short Form 36; HAQ = Health Assessment Questionnaire; DAS28 = Disease Activity Score in 28 joints; DMARDs = disease-modifying antirheumatic drugs.

† *P* < 0.05.

‡ Includes any of hypertension, angina, ischemic heart disease, stroke, pulmonary fibrosis, asthma, chronic obstructive pulmonary disease, diabetes mellitus, thyroid disease, peptic ulcers, hepatic disease, renal disease, demyelinating disease, epilepsy, depression, tuberculosis, or cancer.

§ C-reactive protein level >20 mg/liter or erythrocyte sedimentation rate >28 mm/hour.

In the multivariate analysis, there was a statistically significant association between the improvement in SF-36 PCS and the change in DAS28 score at 6 months (β = 2.92 per unit improvement in DAS28; 95% CI 2.10, 3.75). Those patients who were not working due to ill health or disability rather than working at the start of therapy had significantly lower SF-36 PCS scores at 6 months (β = −4.64; 95% CI −7.71, −1.58) (Table 4).

For the change in SF-36 MCS at 6 months, both the univariate and multivariate models found that patients showing improvements in their DAS28 score at 6 months (β = 3.09 per unit improvement in DAS28; 95% CI 2.37, 3.81 and β = 1.31 per unit improvement in DAS28; 95% CI 0.33, 2.29, respectively) experienced improvements in their SF-36 MCS. In the univariate analysis, patients with a longer disease duration (β = 0.18 per year; 95% CI 0.03, 0.34) or receiving concurrent steroids (β = 3.77; 95% CI 0.52, 7.02) showed greater improvements at 6 months, but these findings did not persist when controlling for other potential confounding variables in the multivariate analysis.

## DISCUSSION

This study has shown that, in addition to controlling disease activity, the anti-TNF agents can significantly improve physical disability and QOL during routine clinical use. The greatest improvements were seen in the SF-36 PCS and the HAQ, with smaller improvements observed in the SF-36 MCS. These improvements were attained at 6 months and were maintained thereafter. Imputation of missing variables at followup did not affect the magnitude of the improvements observed. There was no statistically significant difference in the reported improvements among the 3 anti-TNF agents, although modest differences in treatment persistence among them have previously been reported (32).

Unlike for RA, in PsA there are no specific correlates of changes in either HAQ or SF-36 scores with a minimum clinically important difference (MCID). However, using data from RA, the mean clinical difference in HAQ score at 6 months of 0.63 was far greater than the change of 0.22 HAQ units specified as the MCID for RA (33). Likewise, the mean clinical differences at 6 months of 10.2 in the SF-36 PCS and 7.06 in the SF-36 MCS were greater than the MCIDs specified for RA (+4.4 units on the SF-36 PCS and +3.1 units on the SF-36 MCS) (34).

The study has also shown that improvements in the QOL of patients treated with anti-TNF therapies reported in PsA are much higher than those reported in RA and slightly higher than those reported in ankylosing spondylitis (AS) (35). Similarly, a study from the Norwegian DMARD register (36) found larger improvements, after 1 year of anti-TNF therapy, in the QOL of patients with PsA (n = 172) and AS (n = 249) compared with patients with RA (n = 847), which contributed to the better retention of patients with their treatments in the PsA (77.3%) and AS (77.5%) cohorts of the register compared with the RA cohort (65.4%). Our results also correspond with those observed during RCTs involving 1,990 infliximab-treated patients with PsA, AS, and RA, where the greatest improvements from baseline were observed in the physical role and bodily pain components of the SF-36 (19). Furthermore, the same 2 domains of the SF-36 showed the greatest improvements from baseline in PsA patients treated with adalimumab (n = 151) (37). Patients with PsA treated with etanercept (n = 71) have also reported a significant reduction in disability and an increase in functional capacity (38).

It has also been found that improvements in QOL in PsA patients (n = 146) treated with anti-TNF therapies were superior to PsA patients treated with MTX (n = 380) (18). QOL changes from baseline to 6 months were statistically significant in favor of anti-TNF therapy for only 4 (bodily pain, vitality, physical role, and general health) of the 8 domains of the SF-36.

To our knowledge, this is the first study that has reported on those factors that could either predict (measured at baseline) or were associated with (measured at 6 months) improvements in QOL for patients with PsA. The findings suggest that not working due to ill health or disability was associated with negative impacts on the PCS of the SF-36, which may be a further marker for disease severity independent of disease activity. Not surprisingly, improvements in QOL were also significantly associated with improvements in disease activity. Although the simplified disease activity score (DAS28) was originally developed for RA, it has also been shown to perform better than the Psoriatic Arthritis Response Criteria (PsARC) in RCTs of PsA (39), and to be discriminant and responsive in observational cohorts of PsA (40).

Because the BSRBR was originally developed as an RA register, a limitation to the analysis is that there are certain aspects of the patient's PsA that were not captured, such as the Psoriasis Area and Severity Index scores and whether or not there was also axial involvement that may also have influenced improvements in QOL. Second, as data were collected on a 6-month basis, the analysis precludes a more detailed analysis of the time to initial improvement in utility development (41). A Swedish study of anti-TNF use in RA, PsA, and other spondylarthropathies has suggested that utility improvements occur rapidly (within 2 weeks) and were maintained thereafter (41).

Our results reflect current experience in the use of anti-TNF therapies in patients with PsA in the UK. These findings should be considered in context with the guidelines published by the BSR (21) and the National Institute for Health and Clinical Excellence for PsA (42,43). The guidelines suggest a minimum level of disease activity (at least 3 swollen joints and 3 tender joints despite 2 standard DMARDs) and treatment thresholds included the PsARC. However, neither of the guidelines includes thresholds for improvements in QOL. Although there have been previous efforts to develop specific tools to measure QOL in PsA (44,45), these tools have not been introduced to routine practice (46).

The findings in this observational study provide a more solid basis for health economic modeling compared with RCT data due to the greater external validity of the former (47). As policymakers need cost-effectiveness information that is both internally and externally valid, their decisions should be based on data evolving from routine clinical practice. Furthermore, the use of a generic utility instrument (SF-6D) makes comparisons across different diagnoses and future quality-adjusted life year calculations possible. Although criticisms regarding the SF-6D exist that suggest that it may be less acceptable to the patient compared with the EuroQol, with the risk of more incomplete answers, the EuroQol may underestimate utility in less severe disease states (48).

The HAQ was originally developed for RA (49), but has recently been validated for PsA (50). It has been extensively used for measuring physical function in clinical trials and observational studies (51,52), and has been shown to be adequately sensitive to peripheral disease improvements after anti-TNF therapies in PsA (13,17). It is interesting to note that improvements in functional status as measured by the HAQ paralleled the improvements in the QOL as measured by the SF-36 questionnaire, indicating the close relationship between both domains. In RA, it has been shown that, particularly in later disease, the HAQ score is a composite of both a reversible (inflammation) and irreversible (damage) component (53). The significant improvement in HAQ score in the patients in this study, despite a mean disease duration of 12 years, suggests a significant reversible component within the HAQ score in PsA as well.

Further work is required to determine the MCID thresholds in the HAQ and SF-36 that are specific for PsA, because given the impact of skin disease and other extraarticular manifestations, it cannot be assumed that values for RA will be the same for PsA. Equally important is the need to investigate whether subsequent switching of anti-TNF therapies will predict QOL changes. A recent study of the RA cohort within the BSRBR found that the functional status of those who switched treatment improved with a second anti-TNF agent despite failure of the first drug (54). Data from Sweden have demonstrated that QOL improvements during the first and second anti-TNF courses were similar in PsA (41).

In conclusion, improvements in all domains of QOL and functional status seen in RCTs with anti-TNF therapies in PsA can also be seen in routine clinical practice, with improvements in the PCS being greater than that of the MCS. There were no statistical differences between the 3 anti-TNF therapies.

## AUTHOR CONTRIBUTIONS

All authors were involved in drafting the article or revising it critically for important intellectual content, and all authors approved the final version to be submitted for publication. Dr. Ashcroft had full access to all of the data in the study and takes responsibility for the integrity of the data and the accuracy of the data analysis.

**Study conception and design.** Saad, Ashcroft, Watson, Symmons, Noyce, Hyrich.

**Acquisition of data.** Saad, Ashcroft, Watson, Symmons, Noyce, Hyrich.

**Analysis and interpretation of data.** Saad, Ashcroft, Watson, Symmons, Noyce, Hyrich.

## ROLE OF THE STUDY SPONSOR

The British Society for Rheumatology commissioned the Biologics Register as a UK-wide national project to investigate the safety of biologic agents in routine medical practice. The principal investigators and their team have full academic freedom and are able to work independently of pharmaceutical industry influence. All decisions concerning analyses, interpretation, and publication are made autonomously of any industrial contribution. Members of the Manchester team, British Society for Rheumatology trustees, committee members, and staff complete an annual declaration in relation to conflicts of interest.
